# The Polycyclic Polyprenylated Acylphloroglucinol Antibiotic PPAP 23 Targets the Membrane and Iron Metabolism in *Staphylococcus aureus*

**DOI:** 10.3389/fmicb.2019.00014

**Published:** 2019-01-22

**Authors:** Huanhuan Wang, Frank Kraus, Peter Popella, Aslihan Baykal, Claudia Guttroff, Patrice François, Peter Sass, Bernd Plietker, Friedrich Götz

**Affiliations:** ^1^Microbial Genetics, Interfaculty Institute of Microbiology and Infection Medicine (IMIT), University of Tübingen, Tübingen, Germany; ^2^Institut für Organische Chemie, Universität Stuttgart, Stuttgart, Germany; ^3^Genomic Research Laboratory, Division of Infectious Diseases, Geneva University Hospital, Geneva, Switzerland; ^4^Microbial Bioactive Compounds, Interfaculty Institute of Microbiology and Infection Medicine (IMIT), University of Tübingen, Tübingen, Germany

**Keywords:** antibiotic, PPAP 23, mode of action, iron metabolism, Staphylococci, MRSA, VRE

## Abstract

Recently, a series of endo-type B polycyclic polyprenylated acylphloroglucinols (PPAP) derivatives with high antimicrobial activities were chemically synthesized. One of the derivatives, PPAP 23, which showed high antimicrobial activity and low cytotoxicity, was chosen for further investigation of its bactericidal profiles and mode of action. PPAP 23 showed a better efficacy in killing methicillin resistant *Staphylococcus aureus* (MRSA) and decreasing the metabolic activity of 5-day-old biofilm cells than vancomycin. Moreover, *S. aureus* did not appear to develop resistance against PPAP 23. The antimicrobial mechanism of PPAP 23 was investigated by RNA-seq combined with phenotypic and biochemical approaches. RNA-seq suggested that PPAP 23 signaled iron overload to the bacterial cells because genes involved in iron transport were downregulated and iron storage gene was upregulated by PPAP 23. PPAP 23 affected the membrane integrity but did not induce pore formation; it inhibited bacterial respiration. PPAP 23 preferentially inhibited Fe–S cluster enzymes; it has a mild iron chelating activity and supplementation of exogenous iron attenuated its antimicrobial activity. PPAP 23 was more effective in inhibiting the growth of *S. aureus* under iron-restricted condition. The crystal structure of a benzylated analog of PPAP 23 showed a highly defined octahedral coordination of three PPAP ligands around a Fe (3+) core. This suggests that PPAPs are generally capable of iron chelation and are able to form defined stable complexes. PPAP 23 was found to induce reactive oxygen species (ROS) and oxidative stress. Fluorescence microscopic analysis showed that PPAP 23 caused an enlargement of the bacterial cells, perturbed the membrane, and dislocated the nucleoid. Taken together, we postulate that PPAP 23 interacts with the cytoplasmic membrane with its hydrophobic pocket and interferes with the iron metabolism to exert its antimicrobial activity in *Staphylococcus aureus*.

## Introduction

Herbal medicine has been used in healing practice since ancient times. For example, hyperforin, an extract of *Hypericum perforatum*, known as St. John’s wort, has various pharmacological activities, including antidepressant, antitumoral and antimicrobial effects (Barnes et al., 2001). Hyperforin belongs to the large class of PPAPs, which have fascinating chemical structures and biological activities. Owing to the C3 prenyl substituent, hyperforin is sensitive to air and light, and is thus unstable (Liu et al., 2005). The PPAPs can be divided into three types (A, B, and C) depending on the position of the acyl group on the bicyclic core. Type A PPAPs have a C (1) acyl group and an adjacent C (8) quaternary center, type B PPAPs have a C (3) acyl group, and the rare type C PPAPs have a C (1) acyl group and a distant C (6) quaternary center. PPAPs contain a bicycle [3.3.1] nonane-2,4,9-trione core that is substituted with lipophilic side chains. The rigid core serves as a base structure to arrange the substituents in the correct topographical orientation, which is crucial for the biological activities of these compounds (Ciochina and Grossman, 2006; Biber et al., 2011; Yang et al., 2018).

The concept of separating framework construction from framework decoration led to a major breakthrough in the chemical synthesis of distinct *endo*-type B PPAPs (Biber et al., 2011). This synthetic approach laid the foundation for the synthesis of a large number of *endo*-type B PPAP variants (Guttroff et al., 2017). These variants were investigated for their antimicrobial activity and cytotoxicity in comparison with hyperforin and vancomycin. Among the tested variants, PPAP 23 showed high antimicrobial activity against various bacterial Gram-positive species including MRSA and VRE with MIC values ranging from 0.5 to 4 μg/ml. PPAP 23 showed a half maximal inhibitory concentration (IC_50_) of 96 μg/ml for human monocytic HL-60 cells, which was 15-fold less toxic than hyperforin. Moreover, PPAP 23 is photochemically stable (Guttroff et al., 2017). Since the mode of action remains unknown, we chose PPAP 23 to further investigate its antimicrobial activity and underlying mechanisms. To uncover its mode of action, we employed comprehensive approaches including phenotypic, biochemical and transcriptomic analyses. Our study suggested that PPAP 23 interacted with the membrane, inhibited cellular respiration, interfered with the iron metabolism and increased the oxidative stress in *S. aureus*.

## Results

### PPAP 23 Possessed Bactericidal and Anti-biofilm Activity

**FIGURE 1:**
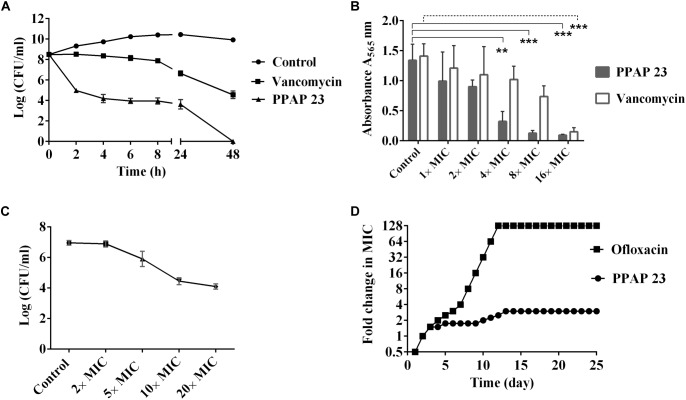
Bactericidal activity, anti-biofilm activity and resistance development of PPAP 23. **(A)** Comparative killing rate of PPAP 23 and vancomycin against *S. aureus* USA300. 100× MIC vancomycin (100 μg/ml) and PPAP 23 (100 μg/ml) were applied to mid-exponential phase *S. aureus* USA300. *S. aureus* without antibiotics was used as control. Samples were taken at the indicated time points, and the number of live cells was determined by the dropping plate assay. Data are mean values of 3 independent experiments ± SD. **(B)** Effect of PPAP 23 on 5-day-old *S. aureus* SA113 biofilm cells. After treatment of 5-day-old *S. aureus* SA113 biofilm with PPAP 23 and vancomycin, the viability of the biofilm cells was determined by the MTT cell proliferation assay. Data are mean values of 3 independent experiments ± SD. Data were analyzed by one-way ANOVA with Dunnett’s multiple comparison with control. Only significant differences are shown with solid line representing PPAP 23 treatment and dashed line for vancomycin treatment, ^∗∗^ indicates *P* < 0.01, ^∗∗∗^ indicates *P* < 0.001. **(C)** Dose-dependent killing assay of *S. aureus* HG001. PPAP 23 at varied concentrations (2×, 5×, 10×, and 20× MIC) were added to inoculum of 10^7^ CFU/ml *S. aureus* HG001 and incubated for 24 h. Error bars stand for standard deviation (*n* = 3). **(D)** Sequential passaging broth assay for the development of spontaneous antibiotic resistance. The *Y*-axis and course of curve represent the antibiotic concentration at which the cells could grow during passaging. Cells treated with ofloxacin rapidly became resistant to a level of 128× MIC (32 μg/ml) whereas successive treatment of *S. aureus* HG001 over 25 days with PPAP 23 resulted in 3× MIC resistance (3 μg/ml). The graph shown in the figure is a single experiment that represents three replicates.

Since PPAP 23 exhibited a comparable MIC to the MRSA USA300 as vancomycin (Guttroff et al., 2017), we then compared its efficacy on killing mid-exponential *S. aureus* USA300 and biofilm cells of *S. aureus* SA 113 with vancomycin. PPAP 23 eradicated the mid-exponential population of *S. aureus* by 8 logs to the limit of detection at 100× MIC in 48 h, whereas the same concentration of vancomycin led to only 3-log reduction in the population of mid-exponential cells (Figure 1A). PPAP 23 was superior to vancomycin in killing the mid-exponential phase *S. aureus*. MTT assay was used to assess the viability of 5-day-old biofilm cells upon exposure to PPAP 23. The yellow tetrazolium MTT [3-(4, 5-dimethylthiazolyl-2)-2, 5-diphenyltetrazolium bromide] is reduced by metabolically active cells to insoluble purple formazan. The quantity of formazan, proportional to the number of viable cells, can be quantified by spectrophotometry. As shown in Figure 1B, PPAP 23 decreased the metabolic activity of *S. aureus* biofilm cells in a concentration-dependent manner. PPAP 23 significantly reduced the metabolic activity of 5-day-old biofilm cells at 4× MIC, whereas vancomycin needed 16× MIC to exert its anti-biofilm activity. This indirect viability assay showed a better efficacy of PPAP 23 on killing established *S. aureus* biofilm cells than vancomycin. PPAP 23 killed an inoculum of 10^7^ CFU/ml *S. aureus* HG001 in a dose dependent manner. The killing rate of PPAP 23 increased from 1- to 3- log reduction as concentrations of PPAP 23 went from 5× to 20× MIC (Figure 1C).

### PPAP 23 Did Not Appear to Lead to Resistance in *S. aureus*

To determine whether PPAP 23-resistant colonies can be generated in *S. aureus* HG001, the cells were serially passaged in TSB medium with daily incremental increases in the concentration of PPAP 23 over a period of 25 days. The maximal concentration of PPAP 23 in which *S. aureus* could grow was 3× MIC = 3 μg/ml (Figure 1D). *S. aureus* grew at 3× MIC were passaged on antibiotic free TSA plates and the MIC was assessed by broth microdilution. No mutants resistant to PPAP 23 were obtained. In contrast, with the gyrase inhibitor ofloxacin (Sato et al., 1986), *S. aureus* cells acquired a resistance of 128× MIC (32 μg/ml) after 10 days. In a single step resistance assay, no resistant colonies were detected on TSA plates containing 2.5× or 5× MIC PPAP 23 after 48 h, suggesting that the frequency of resistance is <10^-10^, whereas *S. aureus* grew in the plates containing 2.5× and 5× MIC ofloxacin in 48 h with mutation frequency of 10^-7^. There were no colonies on plates with 10× MIC PPAP 23 and ofloxacin respectively.

### PPAP 23 Did Not Depolarize the Bacterial Membrane but Induced ATP Release

**FIGURE 2:**
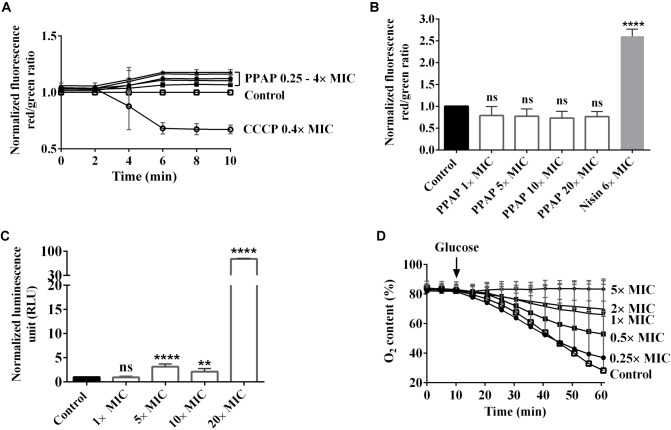
Effect of PPAP 23 on the membrane of *S. aureus*. **(A)** Determination of membrane potential in PPAP 23 treated cells. *S. aureus* HG001 was treated with PPAP 23 at different concentrations, and the relative fluorescence ratios of DiOC2 (3) red/green were calculated. The proton ionophore, CCCP, was used as positive control. **(B)** Detection of membrane permeability and pore formation. Relative fluorescence intensity red/green is shown for *S. aureus* HG001 stained with propidium iodide and SYTO 9 in the presence or absence of PPAP 23 at varied concentrations for 30 min. Nisin was used as the positive control. **(C)** Measurement of ATP release in PPAP 23 treated cells. Relative luminescent signal of *S. aureus* HG001 treated with PPAP 23 at varied concentrations for 3 h was plotted. **(D)** Oxygen consumption was assayed with washed *S. aureus* HG001 cells in an OxoDish^®^ OD24 plate reader. After 10 min preincubation with PPAP 23 at indicated concentrations, respiration was induced by the addition of 1.0 mM glucose and oxygen content (%) in the cells was recorded for 60 min. All data are mean values of 3 independent experiments. Error bars represent standard deviation. Data of **(A–C)** were normalized with the values of control, the untreated cells; Data of **(B,C)** were analyzed by one-way ANOVA with Dunnett’s multiple comparison with control, ns: no significant difference, ^∗∗^*P* < 0.01, ^∗∗∗∗^*P* < 0.0001.

To find out the mode of action, we firstly examined the effect of PPAP 23 on the membrane potential and membrane permeability in *S. aureus* HG001. The carbocyanine dye DiOC2(3) (3,3′-diethyloxacarbocyanine iodide) was used for measuring membrane potential. With increasing membrane potential, the green fluorescent dye self-associates to form red fluorescent aggregates. The spectral shift is proportional to the proton gradient intensity (Shapiro, 2008). As control, we used CCCP (carbonyl cyanide 3-chlorophenylhydrazone) that eliminates the proton gradient thus decreases the membrane potential (Kasianowicz et al., 1984). CCCP rapidly depolarized membrane potential at 0.4× MIC. At varied concentration from 0.25× to 4× MIC, PPAP 23 did not cause depolarization of the membrane in *S. aureus* HG001 as indicated by an increase in the DiOC2(3) red to green ratio (Figure 2A).

Cell membrane permeability was monitored by measuring the fluorescence of the green-fluorescent nucleic acid stain, SYTO 9 and the red-fluorescent nucleic acid stain, propidium iodide. SYTO 9 stains cells with intact membranes and propidium iodide penetrates bacteria with damaged membranes. Nisin, a pore-forming lantibiotic that decreases the SYTO 9 fluorescence was used as a positive control (van Kraaij et al., 1998). Up to a concentration of 20× MIC (20 μg/ml) PPAP 23 did not cause a significant change in the membrane permeability of *S. aureus* HG001, while nisin significantly increased the permeability at 6× MIC (Figure 2B). This result indicated that PPAP 23 did not form pores in the bacterial membrane as Nisin did.

The amount of ATP was measured using a luciferase-based assay. The luciferin reaction is catalyzed by luciferase in the presence of ATP, and the resulting luminescent signal is proportional to the amount of ATP present. As shown in Figure 2C, PPAP 23 caused ATP release in *S. aureus* HG001 starting from 5× MIC and an extensive leakage of ATP was observed at 20× MIC. Taken together, treatment with PPAP 23 at high concentration of 20× MIC did not lead to pore formation but significant ATP release, suggesting that the cytoplasmic membrane integrity was affected so that the small molecules such as ATP (molecular weight: 507 g/mol) was released out.

### PPAP 23 Inhibited Oxygen Consumption in *S. aureus*

Respiration was induced in *S. aureus* HG001 by addition of 1 mM glucose, and oxygen consumption was determined using an OxoDish^®^ OD24 plate reader. PPAP 23 inhibited the oxygen consumption in a concentration dependent manner (Figure 2D). Even at the subinhibitory concentration of 0.5× MIC, PPAP 23 caused a marked inhibition of oxygen consumption. At 5× MIC, oxygen consumption was completely inhibited. This result suggested that PPAP 23 disrupted the respiratory chain in *S. aureus*.

### Comparative Transcriptome Analysis by RNA-seq

To identify potential genes/pathways targeted by PPAP 23, we performed RNA-seq on *S. aureus* HG001 treated with a subinhibitory concentration of PPAP 23 (0.25× MIC) as well as an untreated control. Samples were taken after 10-, 30-, 60-, and 90-min treatment with PPAP 23 for RNA extraction and purification. Genes that were up or down regulated more than 4-fold and that appeared in more than three time points are listed in Table 1. RNA-seq revealed that most of the down-regulated genes in the PPAP 23-treated cells were involved in iron acquisition (Table 1). These genes included: *mntABC* (*sitABC*), iron–manganese ABC transporter genes (Cockayne et al., 1998; Horsburgh et al., 2002); *sstA*, *sstB*, iron ABC transporter sstABCD system permease (Morrissey et al., 2000); *sstC*, iron ABC transporter sstABCD system ATP-binding protein; *isdC*, iron-regulated surface determinant protein gene, which is part of the heme uptake system (Mazmanian et al., 2003; Grigg et al., 2007); *sirA*, iron ABC transporter SirABC system periplasmic binding protein (Heinrichs et al., 1999); *sbnA*, encoding an enzyme required for synthesis of staphyloferrin B (siderophore) (Kobylarz et al., 2016). In contrast, the ferritin gene (*ftnA*), the iron-storage protein was upregulated (Morrissey et al., 2004). There were two more upregulated genes related to the cell wall: *gntK*, encoding gluconate kinase (Kohanski et al., 2007; Riordan et al., 2007) and *murQ*, encoding gluconate kinase *N*-acetylmuramic acid-6-phosphate etherase (Uehara et al., 2006; Borisova et al., 2016). As many of the iron uptake systems were downregulated by elevated iron concentration, we hypothesized that PPAP 23 led to intracellular iron accumulation. This was in line with upregulation of the iron-storage protein, ferritin. The next question was how PPAP 23 caused such a hypothetical elevated level of intracellular free iron. Since PPAP 23 inhibited respiration and some of the respiratory components have bound iron, we then checked the possibility of PPAP 23 interacting with such proteins.

**Table 1:** The most up- and down-regulated genes in the PPAP 23 challenged *S. aureus* HG001.

ORF	Gene description	Fold change

	Down-regulated^a^	
**Iron metabolism**		
SAOUHSC_00636	Iron–manganese ABC transporter MntABC (SitABC) system permease protein (*mntB*)	-4
		
SAOUHSC_00637	Iron–manganese ABC transporter MntABC (SitABC) system ATP-binding protein (*mntA*)	-4
		
SAOUHSC_00746	Iron ABC transporter SstABCD system permease (*sstA*)	-7.7
SAOUHSC_00747	Iron ABC transporter SstABCD system permease (*sstB*)	-10
SAOUHSC_00748	Iron ABC transporter SstABCD system ATP-binding protein (*sstC*)	-5
SAOUHSC_01082	Iron-regulated surface determinant protein (*isdC*)	-4
SAOUHSC_00074	Iron ABC transporter SirABC system periplasmic binding protein (*sirA*)	-4
		
SAOUHSC_00075	2,3-diaminopropionate biosynthesis protein (*sbnA*)	-10

	**Up-regulated^a^**	

**Iron metabolism**		
SAOUHSC_02108	Ferritin (*ftnA*)	4.4
**Cell wall**		
SAOUHSC_02808	Gluconate kinase (*gntK*)	17.4
SAOUHSC_00157	*N*-acetylmuramic acid-6-phosphate etherase (*murQ*)	6.4
**Unknown**		
SAOUHSC_01920	Putative lipoprotein	5.2


*^*a*^The selected genes represent the highly regulated genes with a cut-off fold of 4 appearing in more than three time points.*

### PPAP 23 Inhibited Preferentially the Activities of Iron-Sulfur (Fe–S) Cluster Enzymes

**FIGURE 3:**
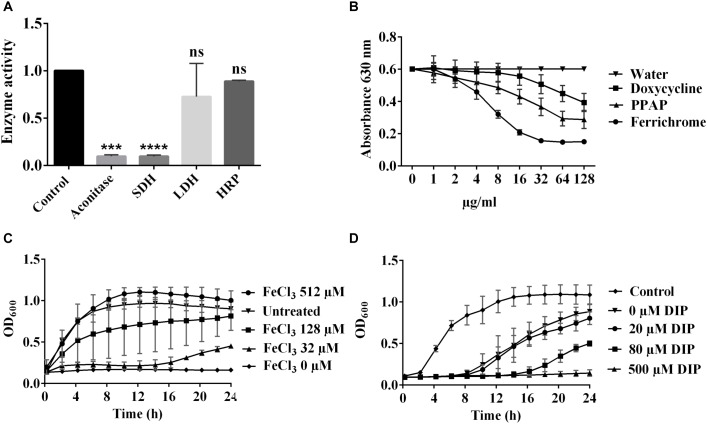
Relation of PPAP 23 and iron. **(A)** Effect of PPAP 23 on Fe–S cluster enzymes. Early exponential phase *S. aureus* HG001 cells were incubated with 1× MIC PPAP 23 for 6 h and cell-free extracts were used for the enzymatic assays of aconitase, SDH and LDH. Effects of PPAP 23 on HRP was determined by the oxidized ABTS formation that can be measured at A405 nm. SDH, succinate dehydrogenase; LDH, lactate dehydrogenase; HRP, horseradish peroxidase. **(B)** Determination of iron chelating activity with the chrome azurol sulfate (CAS) assay. The iron chelating activity is indicated by a reduction of the blue color (A_630_). Various concentrations of PPAP 23, doxycycline and ferrochrome were incubated with the CAS shuttle solution and the color reduction at A_630_ was monitored. **(C)** Effect of exogenous iron on the antimicrobial activity of PPAP 23. Early exponential *S. aureus* HG001 was cultivated in the premixture of 4.6 μM PPAP 23 (2× MIC) and FeCl_3_ of 32, 128, and 512 μM. The growth curves were monitored in the BioTek^TM^ microplate spectrophotometer for 24 h. **(D)** Growth curve of PPAP 23-treated *S. aureus* under iron-restricted condition. Early exponential *S. aureus* HG001 was grown in TSB supplemented with 0.5× MIC PPAP 23 and varied concentrations of 2,2′-dipyridyl (DIP). Optical densities of cultures were determined in the BioTek^TM^ microplate spectrophotometer. All data represent mean values of three independent experiments ± SD. Data of **(A)** were normalized to the untreated control and analyzed by one-way ANOVA with Dunnett’s multiple comparison with control, ns, no significant difference, ^∗∗∗^*P* < 0.001, ^∗∗∗∗^*P* < 0.0001.

Enzymatic assay of cell-free extracts of exponential *S. aureus* HG001 revealed that the Fe–S cluster containing enzymes, aconitase and succinate dehydrogenase (SDH), lost about 90% activity by the treatment of 1× MIC PPAP 23, while the activity of lactate dehydrogenase (LDH) was not significantly inhibited. The activity of heme enzyme horseradish peroxidase (HRP) was not affected by PPAP 23 (Figure 3A). This result indicated that PPAP 23 impaired the activity of Fe–S cluster enzymes more than the non-Fe–S cluster enzyme and heme enzyme, which raised the question whether PPAP 23 was able to interact with iron.

### PPAP 23 Chelated Iron From the CAS-HDTMA-Fe^3+^ Complex

To test whether PPAP 23 interacts with iron due to its chelating property, we deployed the chrome azurol sulfate (CAS) colorimetric assay. The complex formed by CAS, HDTMA and Fe^3+^ produces a blue color. If iron is removed, the blue color will be reduced. This is exactly what we observed when we incubated the CAS/HDTMA/Fe^3+^ complex with PPAP 23. With increasing concentration of PPAP 23, the A_630 nm_ decreased continuously. As controls we also tested the siderophore ferrichrome, and the antibiotic doxycycline that was shown to have iron-chelating activity (Grenier et al., 2000). The iron-free ferrichrome has the highest iron-chelating activity, followed by PPAP 23 and doxycycline (Figure 3B). PPAP 23 and doxycycline at a concentration of 64 μg/ml (145.25 μM for PPAP 23 and 144 μM for doxycycline) reduced the absorbance (A_630_) by approximately 50 and 33%, respectively; while with ferrochrome a 50% decrease was already achieved at 8 μg/ml (11.63 μM). The result showed that PPAP 23 had a mild iron-chelating activity similar to doxycycline.

### Antimicrobial Activity of PPAP 23 Was Weakened in the Presence of Exogenous Iron

When early exponential *S. aureus* HG001 cells were cultured in the premixture of 4.6 μM PPAP 23 (2× MIC) with 32, 128, and 512 μM FeCl_3_, the growth could be restored in a FeCl_3_ dose dependent manner (Figure 3C). FeCl_2_ also attenuated the inhibitory effect of PPAP 23 when it was premixed with PPAP 23 before the bacteria inoculation. But the preincubation of PPAP 23 with other divalent ions such as CaCl_2_, CuCl_2_, MgCl_2_, MnCl_2,_ and ZnCl_2_ at the same molarity could not rescue *S. aureus* (Supplementary Figure S1).

### Antimicrobial Activity of PPAP 23 Was Enhanced in Iron-Restricted Condition

The presence of iron chelator 2,2′-dipyridyl (DIP) induces iron restriction in the medium. Increasing concentrations of DIP from 20, 80, and 500 μM renders *S. aureus* increasingly susceptible to 0.5× MIC PPAP 23 (Figure 3D).

### PPAP 23 Induced ROS Formation

**FIGURE 4:**
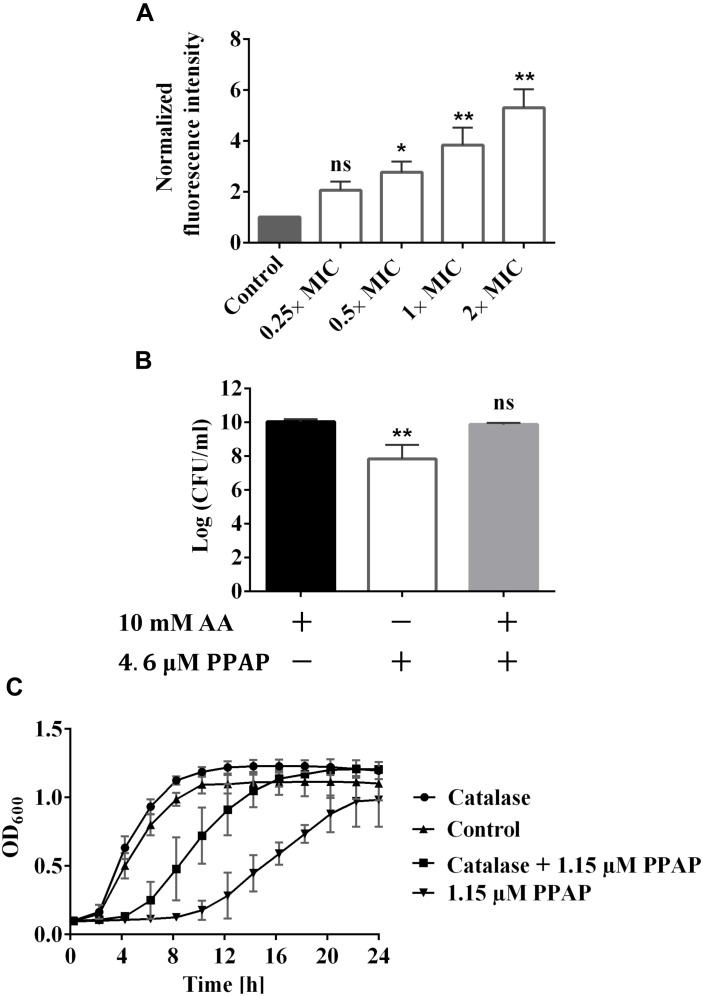
Impact of PPAP 23 on reactive oxygen species (ROS) generation. **(A)** Detection of ROS by DCFH_2_-DA assay. *S. aureus* HG001 cells were stained with 20 μM DCFH_2_-DA for 30 min in the dark before the incubation with PPAP 23 for 3 h. Fluorescence Excitation/Emission were measured at 485 nm/535 nm. **(B)** Enumeration of *S. aureus* HG001 overnight cultures grown in TSA supplemented with 10 mM ascorbic acid (AA), TSA containing 10 mM AA and 4.6 μM PPAP 23, or TSA with 4.6 μM PPAP 23. **(C)** Effect of exogenous catalase (1000 U/ml) on the growth of PPAP 23-treated *S. aureus* HG001. Growth was determined by the optical density in BioTek^TM^ microplate spectrophotometer for 24 h. All data are the mean values of three replicates ± SD. Data of **(A)** were normalized to the untreated control. Data of **(A,B)** were analyzed by one-way ANOVA with Dunnett’s posttest, ns, no significant difference, ^∗^*P* < 0.05, ^∗∗^*P* < 0.01.

Dichlorodihydrofluorescein diacetate (DCFH_2_-DA) is the most widely used probe for detecting intracellular oxidative stress. This probe is cell-permeable and is hydrolyzed intracellularly to the DCFH carboxylate anion, which is retained in the cell. Two-electron oxidation of DCFH by ROS results in the formation of a fluorescent product, dichlorofluorescein (DCF), which was monitored using a Tecan infinity M200 microplate reader. PPAP 23 caused an increase in fluorescence starting from 0.5× MIC concentration and ROS production was PPAP-concentration dependent (Figure 4A). To corroborate the result, we plated serially diluted overnight culture on TSA containing 10 mM of the ROS scavenger, ascorbic acid and 2× MIC PPAP 23. *S. aureus* recovered about 2 logs in the presence of ascorbic acid, indicating ROS production was involved in PPAP 23 treatment (Figure 4B). In addition, excess catalase (1300 U/ml) partially protected *S. aureus* against the inhibitory effect of PPAP 23 at 0.5× MIC (Figure 4C). Since catalase detoxifies H_2_O_2_ to water and oxygen, this result suggested that hydrogen peroxide was involved during PPAP 23 treatment. Taken together, these results suggested that PPAP 23 treatment resulted in ROS production in *S. aureus*.

### PPAP 23 Increased Cell Size, Disintegrated the Membrane and Impaired Nucleoid Organization of *S. aureus*

**FIGURE 5:**
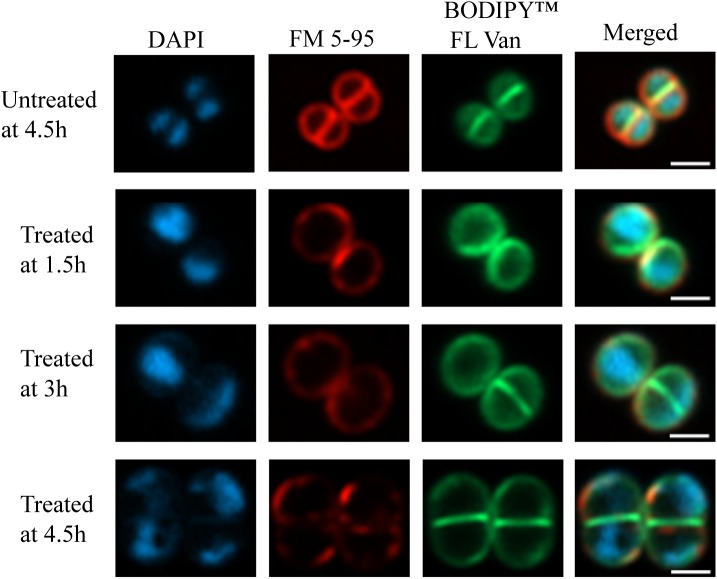
Super-resolution fluorescence microscopy of PPAP 23 treated *S. aureus*. Fluorescent images of untreated *S. aureus* HG001 at 4.5 h and 2× MIC PPAP 23 treated cells at 1.5, 3, and 4.5 h. Cells were labeled with (left to right) DAPI (nucleoid), FM 5-95 (membrane) and BODIPY^TM^ FL vancomycin (cell wall) and merged. The scale bar is 1 μm.

Early exponential cells of *S. aureus* HG001 were treated with 2× MIC PPAP 23 (2 μg/ml); DMSO (1%) was used as a control. In Figure 5, the merged and single-channel fluorescence images showed severe effects of PPAP 23 on cellular substructures. Here, treated cells clearly enlarged in size (∼2-fold in volume) after 4.5 h of PPAP 23 treatment compared to control cells. DAPI staining showed that the bacterial DNA appeared to be dislocated to the periphery of the cells indicating severe disorganization of nucleoids and DNA damage. Also, membrane staining using FM 5-95 showed that the control cells were much more intensively and evenly stained than the treated cells which may be due to a perturbed membrane environment and/or biosynthesis. This phenomenon is either due to a direct effect of PPAP 23 treatment or secondary effects upon antibiotic treatment. BODIPY^TM^ FL vancomycin preferably stained the cross wall (septum) in both treated and untreated cells.

### The X-ray Structure of a Fe^3+^ – PPAP Analog Revealed a [Fe (PPAP)_3_]-Complex

**FIGURE 6:**
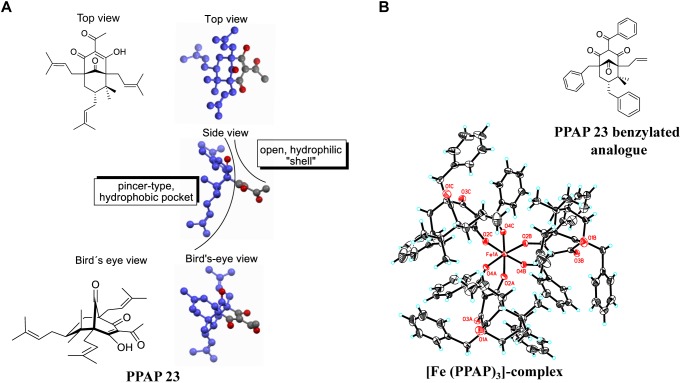
**(A)** 3D-structure of PPAP 23 showing the hydrophobic pocket (dark blue) and the hydrophilic shell; the gray atoms belong to the hydrophilic substructure, and the oxygen atoms of the four carbonyl functionalities are highlighted in red. **(B)** crystal structure of an Fe- PPAP complex showing a highly defined octahedral coordination of the 1,3-diketone motif to an Fe(III)-center. The iron in the middle (Fe1A) and the oxygen atoms are highlighted in red.

A PPAP-based, highly defined metal complex is depicted in Figure 6B. The crystals required for X-ray analysis were obtained by slow evaporation of a dichloromethane solution of the purified Fe-PPAP-complex. The PPAP ligand shown is an analog of PPAP 23 that contains aromatic side chains instead of multiple isoprenyl-residues, which were introduced for the sole purpose to enhance the likeliness of crystallization. Three PPAP ligands organize their 1,3-diketone motifs around an Fe-center that underwent oxidation during the complex formation, since Fe (OAc)_2_ has been used as the iron source. The bond lengths of the iron-oxygen bonds range from 1.95 to 2.00 Å, whereas the bond angles of each O-Fe-O bond are between 84.91 and 85.77 for the O-Fe-O angles that are spanned up by two oxygens of the same PPAP ligand. Bond angles of O-Fe-O bonds between different PPAP ligands are in a range of 89.48 to 93.33 for oxygens located rectangular and 173.03 to 176.75 for oxygen atoms located on opposite sides of the octahedron. These values clearly show a highly defined orientation within the complex. In the experiment the corresponding PPAP was dissolved in methanol and iron (+2) acetate was added at room temperature, which immediately resulted in the formation of a red solution. The obtained product was oxidized to a Fe (+3)-component, which was stable upon extraction into organic solvents, as well as column chromatography on silica gel. Although this reaction setup does not resemble the biological system, the result proofs the general metal chelating properties of PPAPs, as well as the possibility to undergo redox-chemistry under appropriate conditions.

## Discussion

In this study we mainly focused on the investigation of the antimicrobial mechanism of PPAP 23 in our model organism *S. aureus* HG001. At first, we tested whether PPAP 23 had an effect on the bacterial cell envelope, cytoplasmic membrane and/or cell wall. Therefore, we investigated the impact of PPAP 23 on bacterial membrane potential, permeability, ATP release and respiration. PPAP 23 did not cause a depolarization of the cytoplasmic membrane in *S. aureus*, nor did it form pores. However, PPAP 23 induced an extensive leakage of ATP and inhibited oxygen consumption, suggesting that it targets the cytoplasmic membrane and respiration. This was corroborated by the fluorescence microscopic analysis showing a time-dependent decrease of the membrane staining and increase of the cells size (Figure 5). The enlargement of the cells speaks in favor that the PPAP 23 treated cells cannot withstand the osmotic pressure in liquid medium. Peptidoglycan staining with BODIPY^TM^ FL vancomycin showed no gross alterations in the fluorescence images. However, we speculate that peptidoglycan biosynthesis may be impaired as a consequence of the membrane disruption. One evidence that peptidoglycan biosynthesis might also be affected is presented by the RNA-seq showing that *murQ* (MurNAc-P etherase) gene expression was increased in the antibiotic-treated cells (Table 1). MurQ is a *N*-acetylmuramic acid-phosphate (MurNAc-P) etherase that converts MurNAc-6-phosphate to GlcNAc-6-phosphate (Uehara et al., 2006). It stabilizes the cell wall during stationary growth phase by recycling the cell wall sugar MurNAc to enhance survival of *S. aureus* (Borisova et al., 2016). The activation of the *murQ* suggests that peptidoglycan recycling is involved in rescuing PPAP 23 cell wall stress. The 3D structure of PPAP 23 is shown in Figure 6A. As can be seen from the side view the structure exhibits a rigid pincer-type topology with a lipophilic pocket (shown in dark blue) and an open carbonyl-rich hydrophilic shell. The lipophilic pocket of PPAP 23 might interact with the membrane through interrogation of the polyunsaturated aliphatic substituents which could explain the observed altered membrane integrity. These results suggest that the primary target of PPAP 23 is most likely the bacterial membrane.

RNA-seq provides a hint that PPAP 23 also interferes with the iron metabolism. PPAP 23 downregulated genes involved in iron transport, while upregulated the ferritin gene (*ftnA*) encoding iron storage protein (Table 1). The upregulation of *ftnA* and downregulation of iron transporters are suggestive of an excess of intracellular free iron. Further investigation showed that Fe–S cluster enzymes, aconitase and succinate dehydrogenase were inhibited by PPAP 23 treatment, whereas the activity of non-Fe–S cluster enzymes and the heme peroxidase were not significantly affected by PPAP 23 (Figure 3A). The inhibition of succinate dehydrogenase that is part of the respiratory chain could contribute to the oxygen consumption inhibition in *S. aureus* (Figure 2D). The inhibition of the Fe–S cluster enzymes led us to check the Fe-chelating activity of PPAP 23. CAS assay showed that PPAP 23 chelated iron from the CAS-HDTMA-Fe^3+^ complex, and its iron chelating was not as strong as ferrichrome (Figure 3B). Pre-incubation of PPAP 23 with either FeCl_3_ or FeCl_2_ mitigated its inhibitory effect in an iron-concentration-dependent manner (Figure 3C and Supplementary Figure S1A). Moreover, the bacterial growth inhibition by PPAP 23 was strengthened in the iron-restricted medium containing 2,2′-dipyridyl (Figure 3D). We therefore postulate that PPAP 23 exerts its antimicrobial activity in the iron-free form; PPAP 23 could bind to Fe-ions and that this binding neutralizes its antimicrobial activity. The hydrophilic shell of PPAP 23 shown in Figure 6A is most likely involved in the chelating Fe^3+^ since the 1,3-diketone motif is known to be a potent bidentate ligand for Fe (+3)-salts (Tachikawa et al., 2018). The iron chelating capability was further confirmed by the crystal structure of Fe^3+^ bound PPAP 23 analog (Figure 6B). The structural analysis revealed that three PPAP ligands organized their 1,3-diketone motifs around an Fe-center that underwent oxidation during the complex formation. However, it remains unclear what the conformation of a putative iron-complex will be in the living cell system, but it is rational to assume that PPAP could bind to iron of the Fe–S cluster enzymes in the cells.

**FIGURE 7:**
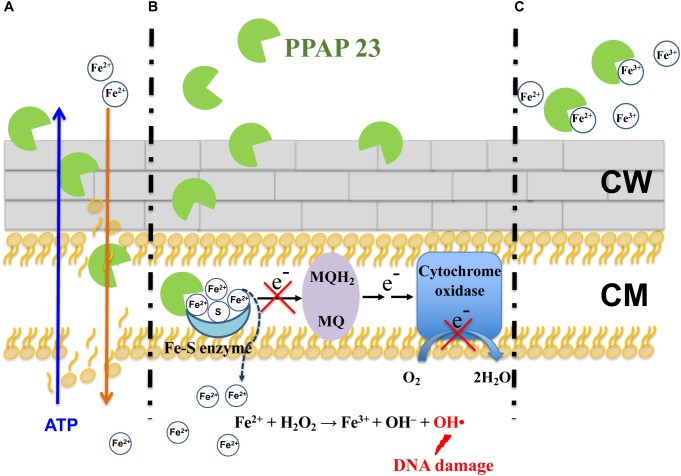
Proposed antimicrobial model of PPAP 23 in *S. aureus*. **(A)** PPAP 23 interacts with the membrane with its lipophilic pocket thus affecting the membrane integrity. It is likely that ATP molecules diffuse out and iron diffuse into the cells through the distorted membrane. **(B)** PPAP 23 chelates iron from Fe–S cluster enzymes resulting in the inactivation of Fe–S cluster enzyme. This has two effects: First, if Fe–S cluster enzymes are involved in the respiration (for example succinate dehydrogenase), respiration will be disturbed. Second, the labile iron may be dissociated from the destabilized enzyme. The released iron makes cells signal iron overload and stimulate Fenton reaction resulting in DNA damage and ultimate cell death. **(C)** PPAP 23’s antimicrobial activity is alleviated when it is bound with iron. MQH2, menaquinol; MQ, menaquinone; CW, cell wall; CM, cytoplasmic membrane.

Regarding how PPAP 23 mediates an increase of free iron in the cytoplasm as suggested by RNA-seq, one possibility is that PPAP 23 causes membrane distortion to allow uncontrolled iron diffusion into the cells; another possibility is that PPAP 23 chelates the iron from the Fe–S cluster enzymes and destabilizes enzymes, iron is then liberated from the destabilized enzymes into the cytoplasm as illustrated in Figures 7A,B. Either case would lead to an overload of free iron in the cell, which could favor the Fenton Reaction to produce highly reactive hydroxyl radicals (Walling, 1975). Indeed, using the DCFH2-DA probe assay, we detected an increased level of ROS in *S. aureus* when exposed to PPAP 23 (Figure 4A). This result was corroborated by the finding that ROS scavenger ascorbic acid could counteract the antibacterial activity of PPAP 23 to a certain extent (Figure 4B) and that catalase partially alleviated the antimicrobial effect of PPAP 23 (Figure 4C). Hydroxyl radicals are highly reactive and readily modify and break DNA, resulting in DNA damage and eventual cell death (Aruoma et al., 1991). As shown by fluorescence microscopy, severe DNA disorganization was induced by PPAP 23 treatment (Figure 5). When the ROS level exceeds the antioxidant capability of the bacteria, oxidative stress will occur (Gutteridge and Halliwell, 2018). The results suggest that PPAP 23 treatment leads to oxidative stress in *S. aureus*. Another indication for the oxidative stress is that PPAP 23 induced upregulation of the gluconate kinase gene (*gntK*) in *S. aureus* (Table 1). The gluconate operon (*gntRKP*) is involved in pentose phosphate pathway, which generates NADPH. It has been reported that NADPH is involved in the cellular response to oxidative stress (Pomposiello et al., 2001; Gaupp et al., 2012). We therefore hypothesize that PPAP 23 induces ROS formation and oxidative stress like many other bactericidal antibiotics (Pomposiello et al., 2001; Kohanski et al., 2007).

*Staphylococcus aureus* is well equipped with siderophore-mediated iron and heme uptake systems to acquire iron from the extracellular environment (Ballouche et al., 2009; Hammer and Skaar, 2011). It is unlikely that PPAP 23 utilizes one of the transport systems to enter the cells, otherwise resistant mutants would have been obtained. PPAP 23 is prone to interaction with the cytoplasmic membrane with its lipophilic pocket. We provide various evidences that PPAP 23 kills bacteria in the iron-free form by disintegrating the cytoplasmic membrane and interfering with the iron metabolism. It remains unknown as to why the cells sensed an overload of intracellular free iron upon exposure to PPAP 23. We speculate that the disintegration of the cytoplasmic membrane could facilitate iron influx as it aids ATP efflux, and it is also possible that iron may dissociate from the inactivated Fe–S cluster enzymes. The abundance of cytoplasmic iron could fuel iron-based Fenton reaction to produce deleterious hydroxyl radicals that break DNA leading to ultimate cell death (Figures 7A,B). When PPAP 23 is bound with extracellular iron in the medium, its antimicrobial activity is attenuated (Figure 7C).

PPAP 23 has potent activity against multiple resistant Gram-positive pathogens and low risk of developing detectable resistance, which makes it a promising antibiotic. Further deciphering its mode of action paves the way for designing new PPAP derivatives with potential use in the treatment of infections by multidrug-resistant bacteria.

## Materials and Methods

### Bacterial Strains and Growth Conditions

The strains used in the experiments were *S. aureus* USA300, *S. aureus* SA113 and *S. aureus* HG001. *S. aureus* was grown in Tryptic Soy Broth (TSB, Difco) to the desired phase as specified in each experiment. For the determination of MIC, Mueller Hinton Broth (MHB) was used as medium.

### Minimal Inhibitory Concentration (MIC) Determination

The MIC was determined by the microdilution method according to the guidelines of the Clinical and Laboratory Standards Institute document M07-A9 (CLSI, 2012). Antibiotics were serially diluted (32 μg/ml to 0.06 μg/ml) in 96-well microplates. Equal volumes of the bacterial inoculum 1× 10^6^ CFU/ml were added, and the cultures were incubated at 37°C under continuous shaking for 18 h. The MIC was determined as the lowest concentration that completely inhibited visible growth of the bacterium. PPAP 23 has the same MIC value of 1 μg/ml (2.3 μM) for *S. aureus* USA300, SA113 and HG001. Unless stated otherwise, control refers to bacterial cells treated with DMSO at a concentration equivalent to the highest concentration added in PPAP treated samples.

### Killing Assay

Methicillin resistant *S. aureus* USA300 is a multiple resistant strain that is not only resistant to the entire class of β-lactam antibiotics but also a number of other common antibiotics making MRSA infection difficult to cure (Diep et al., 2006; Otto, 2012). Antibiotics that are able to slay USA300 are of particular clinical interest. Therefore, we used this strain to test the killing effect of PPAP 23. Overnight culture of *S. aureus* USA300 was diluted 1:10,000 in fresh TSB medium and incubated at 37°C with aeration at 150 r.p.m. for 4 h till mid-exponential phase. Vancomycin and PPAP 23 were then added to a final concentration of 100× MIC (100 μg/ml). *S. aureus* USA300 without antibiotic treatment was included as the control. Samples were taken at intervals of 2, 4, 6, 8, 24, and 48 h, and serially diluted in sterile phosphate buffered saline (PBS). The viable bacterium in serial dilution were determined by the drop plate method (Herigstad et al., 2001). Overnight culture of *S. aureus* HG001 was diluted with TSB to achieve a starting inoculum of approximately 10^7^ CFU/ml and challenged with varied concentrations of PPAP 23 (2×, 5×, 10×, and 20× MIC) for 24 h. CFU per ml were calculated from the spotted serial dilution on tryptic soy agar plate after overnight incubation at 37°C.

### Biofilm Assay

*Staphylococcus aureus* SA113 forms strong biofilm (Cramton et al., 1999) and therefore was used in the biofilm assay. MTT assay was employed as the indirect viability determination (Saising et al., 2012). Overnight culture of *S. aureus* SA113 was diluted to OD_578_ = 0.1, and 100 μl of the inoculum was added to wells of a U-bottom microplate and incubated at 37°C for 5 days. The medium was removed, and the wells were rinsed twice with PBS. 200 μl PPAP 23 was then added at concentrations of 1×, 2×, 4×, 8×, and 16× MIC and incubated at 37°C for 24 h. The supernatant was discarded and replaced with 200 μl of PBS supplemented with 50 g MTT (Sigma-Aldrich). The plate was then incubated at 37°C for 1 – 4 h. Formazan crystals were dissolved in dimethyl sulfoxide (DMSO) by incubation at 37°C for 30 min before measuring the absorbance at 565 nm with a microplate reader (TECAN Infinite M200).

### PPAP 23 Resistant Development

The single step resistance and sequential passaging method were used to develop resistant mutants. We used *S. aureus* HG001 as a model strain for the resistant development and the following experiments as it is a *rsbU*-repaired derivative of the well-characterized NCTC 8325 (Herbert et al., 2010; Caldelari et al., 2017). The genome sequence of HG001 is of high quality. For the single step resistance assay (Firsov et al., 2004; Ling et al., 2015), *S. aureus* with inoculum of 10^10^ CFU/ml was plated on TSA plates containing 2.5×, 5×, 10× MIC PPAP 23 and incubated at 37°C for 48 h before the examination of the colonies. For the sequential passaging, overnight culture of *S. aureus* HG001 was diluted to OD_578_ of 0.02 in 2.5 mL TSB containing 0.5× MIC PPAP. Cells were incubated at 37°C with agitation for 24 h. Culture showing visible growth after 24 h was inoculated 1:500 into fresh TSB medium in the presence of PPAP 23 at an incremental increase in concentration. The serial passaging was carried out for 25 days. Final culture at 25 days that grew at higher than the MIC levels were passaged on antibiotic free MHB to determine its MIC by broth microdilution. Ofloxacin was used as a control.

### Membrane Potential Depolarization and Membrane Permeability

The membrane potential assay was conducted using the BacLight^TM^ Bacterial Membrane Potential Kit. Briefly, exponential phase *S. aureus* HG001 was washed with PBS thoroughly and resuspended to achieve OD_578_ = 0.4. 100 μL of the bacterial cell suspension was pipetted into a 96-well flat-bottom microplate. Cells were stained with DiOC2(3) for 30 min in the dark to let the dye load into the cell membrane. The fluorescence intensity was monitored for 2 min using a Tecan infinity M200 microplate reader with an excitation wavelength of 480 nm and emission wavelength of 630 and 530 nm for red and green fluorescence, respectively. 0.25×, 0.5×, 1×, 2×, and 4× MIC PPAP was then added to the bacterial cell suspension mixture and the fluorescence was recorded every 30 s for an additional period of 8 min. CCCP was included as positive control.

Membrane permeability was determined according to the manufacturer’s instructions (LIVE/DEAD^TM^ BacLight^TM^ Bacterial Viability Kit for microscopy and quantitative assays). *S. aureus* HG001 was prepared as described for the membrane potential depolarization assay. 100 μl of *S. aureus* was incubated with an equal amount of the staining solution containing the SYTO 9 dye (10 μM in DMSO solution) and propidium iodide (60 μM in DMSO solution) for 15 min in the dark. The fluorescence was recorded for 5 min in a Tecan infinity M200 microplate reader (excitation wavelength of 485 nm and emission wavelength of 620/530 nm for red/green fluorescence, respectively). PPAP 23 was then added over a range of concentrations (1×, 5×, 10×, and 20× MIC), and the fluorescence was monitored for an additional 30 min. Nisin was used as the positive control.

### ATP Release

PPAP 23 at varied concentrations of 1×, 5×, 10×, and 20× MIC were added to exponential *S. aureus* HG001 and incubated at 37°C with shaking for 3 h. Bacteria were then harvested, and the supernatant was filtered with a 0.22 μm filter (Millipore). The released ATP from bacteria was measured using the BacTiter-Glo^TM^ Microbial Cell Viability Assay kit according to the manufacturer’s instructions.

### Oxygen Consumption

Overnight culture of *S. aureus* HG001 was diluted with TSB to OD_578_ = 0.05 and incubated at 37°C with shaking for 2 h until the cultures were in early exponential phase. Cells were washed thoroughly with PBS and then resuspended to OD_578_ = 0.5. 1ml/well cell suspension was added to the OxoDish^®^ OD24 plate, which contains pre-calibrated oxygen sensors. The cells were incubated with PPAP 23 at varied concentrations (0.25×, 0.5×, 1×, 2×, 5× MIC) for 10 min before the addition of 1 mM glucose to induce respiration and oxygen consumption. Oxygen content in the cells was recorded till 60 min using the SDR SensorDish^®^ Reader (PreSens Precision Sensing GmbH, Germany).

### RNA-Seq

A sample of early exponential phase *S. aureus* HG001 was taken before the addition of PPAP 23 at time point 0 as the baseline. PPAP 23 was then added at the subinhibitory concentration of 0.25 μg/ml (0.25× MIC). Samples were taken after 10-, 30-, 60-, and 90-min incubation with PPAP 23 for RNA extraction by Qiagen RNA purification kit as described by the manufacturer. RNA was extracted from the untreated *S. aureus* HG001 at the same time points in order to compare the changes of gene expression. Purified RNA was treated with DNase I, quantified by using Qubit (Life Technologies), and RNA integrity was assessed with a 2100 Bioanalyzer (Agilent Technologies). One μg of total RNA was ribodepleted with a bacterial Ribo-Zero kit from Illumina. A TruSeq RNA stranded kit from Illumina was used for library preparation. The library quantity was measured with Qubit, and quality was assessed on a Tapestation on a DNA high-sensitivity chip (Agilent Technologies). The libraries were pooled at equimolarity and loaded at 2 nM for clustering. Oriented 50-base single-read sequencing was performed on an Illumina HiSeq 4000 sequencer, yielding a minimum of X million mapped reads per sample. Final RNA-seq analysis and data analysis were carried out using previously described procedures (Kim et al., 2014). Statistical analyses were done in R v3.2.3 using the edgeR package following a bioinformatics protocol described previously. Briefly, genes whose expression was not at least one read per million in two replicates of the samples were filtered out. Counts of the retained genes were normalized according to the weighted trimmed mean of M values (TMM) method. Read counts for each transcript in each sample were modeled according to a negative binomial (NB) distribution. Finally, pairwise comparisons between read libraries from various strains were performed with the exact test (Robinson et al., 2010; Anders et al., 2013) to detect differentially expressed transcripts. *P*-values were corrected according to the false-discovery rate (FDR). The complete RNA-seq data set has been deposited in European Nucleotide Archive (ENA) under accession number PRJEB28806.

### Enzyme Assays

Cell-free extracts of *S. aureus* HG001 were prepared as previously described with slight modifications (Somerville et al., 2003; Reeder et al., 2011). Early exponential phase cells were incubated with 1× MIC PPAP 23 for 6 h. 20 ml cells were then centrifuged, and the resultant pellets were resuspended in 5 ml buffer (20 mM Tris and 100 mM NaCl, pH 7.4). Subsequently, 15 μg/ml lysostaphin was added, and the suspension was incubated at 37°C until a confluent lysis was observed. The lysed cells were passed through a FastPrep-24^®^ instrument (MP Biomedicals) at the speed 6.5 M/S for 30 s with a 5 min ice incubation interval before the second round. After centrifugation at 21,000 *g* for 15 min at 4°C, the supernatant was analyzed for the activity of aconitase, succinate dehydrogenase (SDH), and lactate dehydrogenase (LDH). Aconitase activity was tested in a 96-well plate using the Sigma-Aldrich Aconitase Activity Assay Kit according to manufacturer’s instructions. The conversion of citrate to isocitrate by aconitase was assessed at 450 nm using a Tecan infinity M200 microplate reader. Succinate dehydrogenase activity was determined using the Succinate Dehydrogenase Assay Kit from Sigma-Aldrich according to manufacturer’s instructions. Absorbance at 600 nm, which is proportional to the enzyme activity, was measured in a Tecan infinity M200 microplate reader. LDH activity was determined using the Pierce LDH Cytotoxicity Assay Kit (Thermo Scientific). The amount of LDH is proportional to the absorbance at 490 nm. All values were normalized to control cells and the obtained relative values were plotted.

The activity of horseradish peroxidase (HPR) was measured by the oxidation of ABTS – 2,2′-Azino-bis (3-Ethylbenzthiazoline-6-Sulfonic Acid) by HPR in the presence of hydrogen peroxide. The procedure was described in the Enzymatic Assay of PEROXIDASE (EC 1.11.1.7, Sigma-Aldrich) with slight modifications. 9.1 mM ABTS was freshly prepared in 100 mM potassium phosphate, pH 5.0. HRP (Sigma-Aldrich) was diluted with cold 40 mM Potassium Phosphate Buffer containing 0.25% (w/v) Bovine Serum Albumin and 0.5% (v/v) Triton X-100, pH 6.8 to obtain 0.8 unit/ml HPR. 30% H_2_O_2_ was diluted with deionized water to obtain 0.3% H_2_O_2_. 290 μl ABTS, 5 μl HPR solution of 0.8 unit/ml, and PPAP 23 at final concentration of 2.3 μM in 305 μl reaction were added to 48-well microplate and mixed well. 5 μl enzyme dilute instead of 0.8 unit/ml HPR solution was added to the blank reactions. Absorption at 405 nm was monitored until constant in Tecan infinity M200 microplate reader. 10 μl 0.3% H_2_O_2_ was then added to start the catalysation reaction and the change in absorptance at 405 nm was recorded for 2 min. Reaction without PPAP 23 was the control.

### Chrome Azurol Sulfate (CAS) Assay

Iron-chelating activity of PPAP 23 was measured by the indicators CAS and hexadecyltrimethylammonium bromide (HDTMA). CAS and HDTMA form a tight complex with ferric iron to produce a blue color. When a strong chelator is present, the iron will be removed from the dye complex resulting in a loss of blue color. The color change is recorded at the absorbance 630 nm_._ The CAS assay was conducted as previously described (Schwyn and Neilands, 1987). 0.0219 g of Hexadecyltrimethy lammonium bromide (HDTMA) was dissolved in 50 ml MilliQ water. 4.307 g anhydrous piperazine was dissolved in 30 ml water and its pH was adjusted with concentrated HCl to 5.6. 1.5 ml FeCl_3_ in 10 mM HCl, 7.5 ml 2 mM CAS, and the piperazine buffer solution were added to HDTMA solution while stirring. The CAS solution was brought to a final volume of 100 ml with water. 4 mM sulfosalicylic acid was added to the above CAS solution to obtain the CAS shuttle solution. 100 μl of CAS shuttle solution and equal volume of doxycycline and PPAP 23 were added to the microplate wells and allowed to react for 40 min. Iron-free ferrichrome (siderophore) from *Ustilago sphaerogena* (Emery, 1971) (Sigma-Aldrich) and doxycycline were used as positive controls (Grenier et al., 2000). The absorbance was measured at 630 nm by TECAN Infinite M200 microplate reader.

### Growth of PPAP 23 Treated *S. aureus* in the Presence of Exogenous Iron and Iron-Restricted Condition

2× MIC PPAP 23 (4.6 μM) was added to a 48-well microplate containing varied concentrations of FeCl_3_ at 32, 128, and 512 μM. Early exponential *S. aureus* HG001 was then added to the microplate wells and incubated at 37°C with continuous agitation in BioTek^TM^ microplate spectrophotometer for 24 h. A common iron chelator 2,2′-dipyridyl (DIP; Sigma-Aldrich) was added to the medium at varied final concentrations of 20, 80, and 500 μM to induce iron restriction growth condition. 0.5× MIC (1.15 μM) of PPAP 23 was added simultaneously to early exponential *S. aureus in* 48-well microplate. The plate was incubated at 37°C with shaking for 24 h in BioTek^TM^ microplate spectrophotometer.

### Intracellular Reactive Oxygen Species (ROS) Measurement and ROS Scavenger Agar Assay

Overnight culture of *S. aureus* HG001 were subcultured in fresh TSB to OD_578_ of 0.05 and grown for 4 h to the mid-exponential phase. 1 × 10^6^ cells/well were then added to a black 96-well microplate reader and stained with 20 μM 2′,7′-dichlorofluorescin diacetate (DCFH_2_-DA, Sigma-Aldrich) for 30 min in the dark. After staining, cells were treated with varied concentrations of PPAP 23 for 3 h before measuring fluorescence at excitation and emission wavelengths of 485 and 535 nm respectively using a Tecan infinity M200 microplate reader. The antioxidant ascorbic acid is a ROS scavenger. To confirm the production of ROS in *S. aureus* by PPAP 23, overnight culture of *S. aureus* was serially diluted and plated on TSA plates supplemented with 10 mM ascorbic acid, TSA plates containing 10 mM ascorbic acid and 2× MIC PPAP and TSA plates with 2× MIC PPAP. Colonies was counted after incubation at 37°C overnight. To check the production of H_2_O_2_, overnight culture of *S. aureus* was diluted to OD_578_ of 0.05 with TSB containing bovine liver catalase (1000 U/ml, Sigma-Aldrich) and challenged with 0.5× MIC PPAP 23. Growth was recorded in BioTek^TM^ microplate spectrophotometer at 37°C with continuous agitation for 24 h.

### Super-Resolution Fluorescence Microscopy

Early exponential *S. aureus* cells were incubated with 2× MIC PPAP 23 at 37°C, 150 rpm. Samples were taken at 1.5 h intervals for 4.5 h and were subsequently stained with 10 μg/ml 4′,6-diamidino-1-phenylindole (DAPI, Invitrogen^TM^ Molecular Probes^TM^), 6.6 μg/ml FM 5-95 (Invitrogen^TM^ Molecular Probes^TM^), and 1 μg/ml BODIPY^TM^ FL vancomycin (Invitrogen^TM^ Molecular Probes^TM^) for 5 min to visualize chromosome, membrane and cell wall, respectively (Sass et al., 2011). For microscopy, 0.5 μl of the stained samples were added to microscopy slides covered with a thin layer of 1% agarose. Images were acquired using a Zeiss Axio Observer Z1 LSM800 equipped with Airyscan detector and C Plan-Apo 63x/1.4 Oil DIC objective (Zeiss). Image analysis was performed via ZEN 2.3 image analysis software package (Zeiss).

### X-Ray-Analysis

PPAP (41.5 mg, 0.08 mmol) was dissolved in methanol (2 mL) and Fe(OAc)_2_ (13.9 mg, 0.08 mmol) was added. The red solution was stirred at room temperature for 30 min. Afterward, water (10 mL) was added, which resulted in the precipitation of a red slurry. The solid was extracted into dichloromethane (3 × 10 mL). The combined organic layers were dried over Na_2_SO_4_, filtered and the solvent was evaporated. The crude product was purified by column chromatography (petrolether: ethyl acetate = 10: 1) to yield the product as a red oily substance (23.2 mg, 0.014 mmol, 54%). Fine needles were obtained by slow evaporation from a concentrated dichloromethane solution. The crystals were analyzed by X-ray analysis, for which the following data were obtained.

**Table:** 

Identification code	s2604lm2_sq
Empirical formula	C105 H99 Fe O12
Formula weight	1608.69
Temperature	130(2) K
Wavelength	0.71073 A
Crystal system, space group	Monoclinic, C2/c
Unit cell dimensions	a = 27.3813(11) A alpha = 90°
	b = 14.7668(4) A beta
	= 102.315(3)°
	c = 45.5880(13) A gamma
	= 90°
Volume	18008.6(10) Aˆ3
Z, Calculated density	8 1.187 Mg/mˆ3
Absorption coefficient	0.229 mmˆ-1
F (000)	6808
Crystal size	0.206 × 0.100 × 0.080 mm
Theta range for data collection	1.522 to 25.368 deg.
Limiting indices	-32 ≤ h ≤ 10, -17 ≤ k ≤
	17, -54 ≤ l ≤ 54
Reflections collected/unique	118433/16497 [R(int)
	= 0.0989]
Completeness to theta = 25.242	100.0%
Absorption correction	Semi-empirical from
	equivalents
Maximum and minimum	0.7452 and 0.7033
transmission	
Refinement method	Full-matrix least-squares
	on Fˆ2
Data/restraints/parameters	16497/18/1069
Goodness-of-fit on Fˆ2	1.018
Final R indices [I > 2sigma(I)]	R1 = 0.0512, wR2 = 0.1087
R indices (all data)	R1 = 0.0971, wR2 = 0.1203
Largest diff. peak and hole	0.675 and -0.409 e.Aˆ-3


Selected bond lengths [A] and angles [deg] for s2604lm2_sq.

FE1A-O(2C)	1.9528(17)
FE1A-O(2B)	1.9679(16)
FE1A-O(4A)	1.9756(15)
FE1A-O(4B)	1.9896(17)
FE1A-O(2A)	1.9813(16)
FE1A-O(4C)	1.9995(16)
O(1A)-C(1A)	1.215(3)
O(2A)-C(7A)	1.276(3)
O(3A)-C(9A)	1.215(3)
O(4A)-C(29A)	1.271(3)


O(2C)-FE1A-O(2B)	92.52(7)
O(2C)-FE1A-O(4A)	93.33(7)
O(2B)-FE1A-O(4A)	173.03(7)
O(2C)-FE1A-O(4B)	175.63(7)
O(2B)-FE1A-O(4B)	84.91(7)
O(4A)-FE1A-O(4B)	89.48(7)
O(2C)-FE1A-O(2A)	91.54(7)
O(2B)-FE1A-O(2A)	90.74(7)
O(4A)-FE1A-O(2A)	85.32(7)
O(4B)-FE1A-O(2A)	92.02(7)
O(2C)-FE1A-O(4C)	85.77(7)
O(2B)-FE1A-O(4C)	91.21(7)
O(4A)-FE1A-O(4C)	93.00(7)
O(4B)-FE1A-O(4C)	90.75(7)
O(2A)-FE1A-O(4C)	176.75(7)
C(7A)-O(2A)-FE1A	132.05(16)
C(29A)-O(4A)-FE1A	132.93(16)


## Author Contributions

FG, HW, and PP designed the study. FK, AB, CG, and BP synthesized the antibiotic. PF performed the RNA-seq analysis. PS conducted fluorescence microscopy experiments. FK conducted structure crystallization. HW performed the rest of the experiments. FG and HW wrote the manuscript. BP proofread the manuscript.

## Conflict of Interest Statement

The authors declare that the research was conducted in the absence of any commercial or financial relationships that could be construed as a potential conflict of interest.

## Abbreviations

CFU: colony forming units
MIC: minimal inhibitory concentration
MRSA: methicillin resistant *S. aureus*
MTT: methylthiazoltetrazolium
PPAP: polycyclic polyprenylated acylphloroglucinols
ROS: reactive oxygen species
VRE: vancomycin-resistant enterococci
