# Supplementary material for: The Polycyclic Polyprenylated Acylphloroglucinol Antibiotic PPAP 23 Targets the Membrane and Iron Metabolism in Staphylococcus aureus
Source: Front Microbiol. 2019 Jan 22;10:14. doi: 10.3389/fmicb.2019.00014 (PMC6352742; doi:10.3389/fmicb.2019.00014)
Supplement: Supplementary file 1 [file Data_Sheet_1.docx]

Supplementary Material

**The polycyclic polyprenylated acylphloroglucinol antibiotic PPAP 23 targets the membrane and iron metabolism in *Staphylococcus aureus***


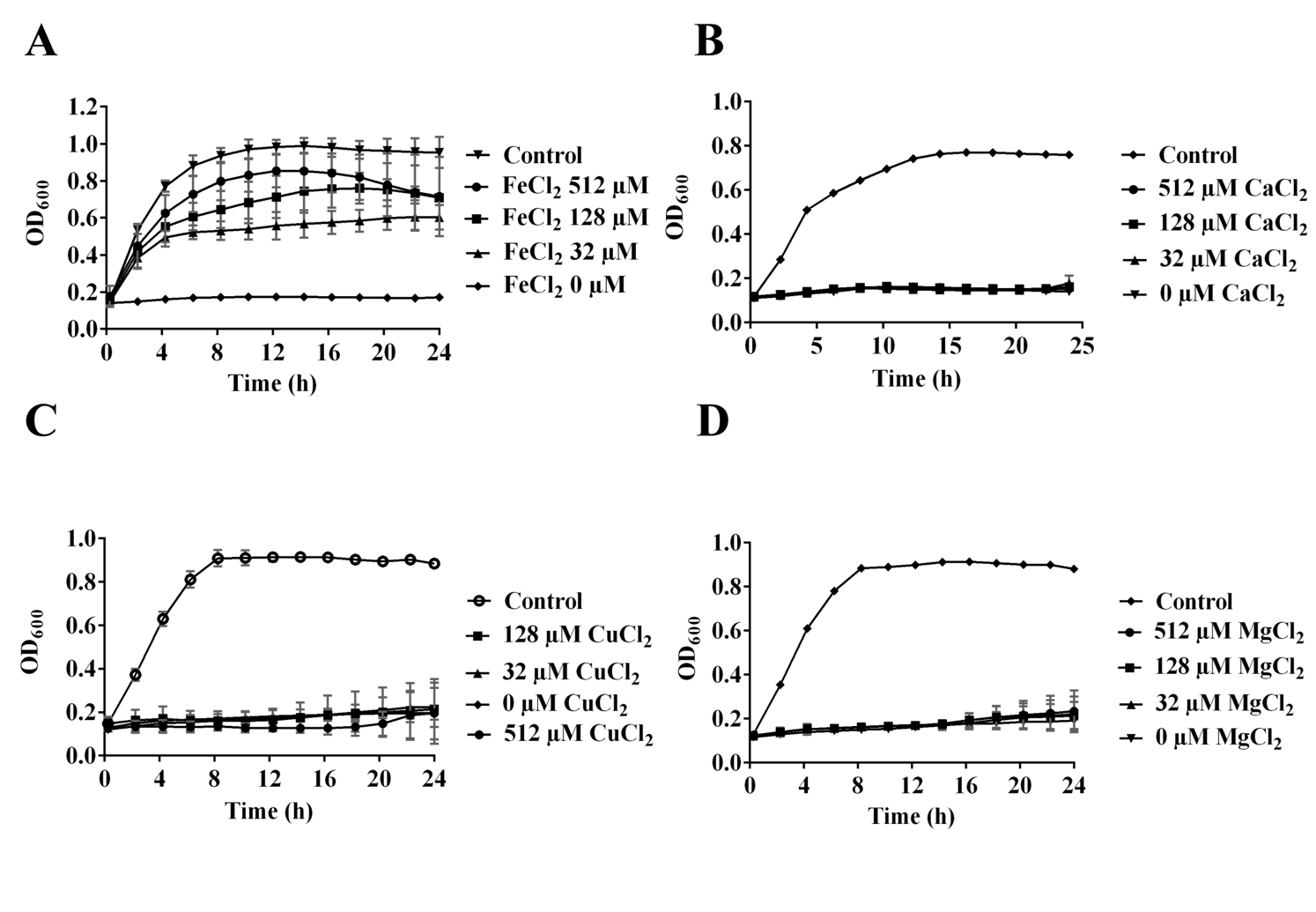

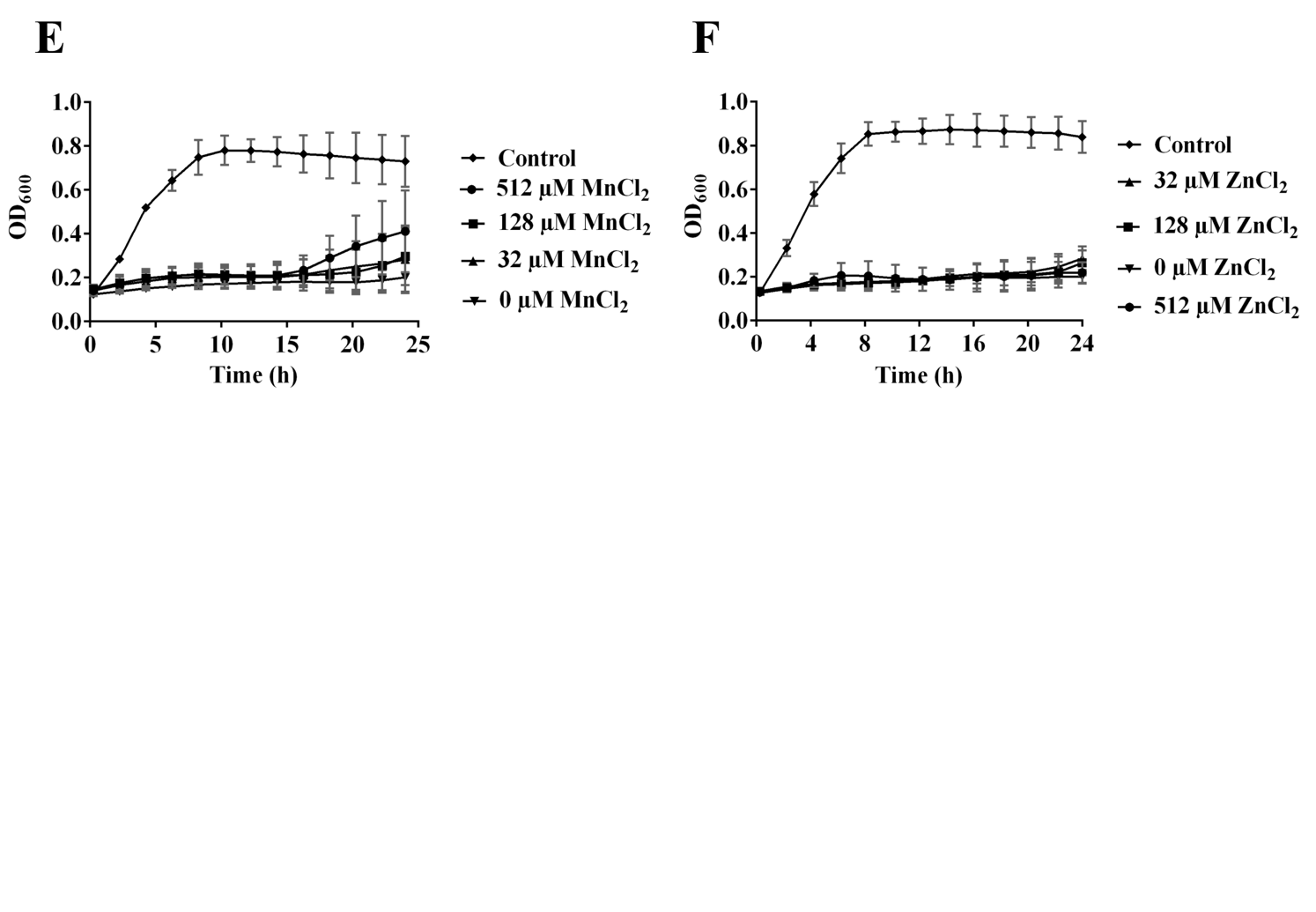


**Supplementary Figure 1.** Effect of FeCl_2_, CaCl_2_, CuCl_2_, MgCl_2_, MnCl_2_ and ZnCl_2_ on the antimicrobial activity of PPAP 23. Early exponential *S. aureus* HG001 was incubated with the premixture of 4.6 μM PPAP 23 (2× MIC) and the indicated irons of 32, 128 and 512 μM. The growth curves were monitored in BioTek™ microplate spectrophotometer for 24 h. Error bars represent the standard deviation (n=3).
